# Plasma pharmacokinetics and synovial concentrations of S-flurbiprofen plaster in humans

**DOI:** 10.1007/s00228-015-1960-6

**Published:** 2015-10-06

**Authors:** Ikuko Yataba, Noboru Otsuka, Isao Matsushita, Miho Kamezawa, Ichimaro Yamada, Sigeru Sasaki, Kazuo Uebaba, Hideo Matsumoto, Yuichi Hoshino

**Affiliations:** Development Headquarters, Taisho Pharmaceutical Co. Ltd, 3-24-1 Takada, Toshima-ku, Tokyo, 170-8633 Japan; Mitsubishi Tanabe Pharma Corporation, Tokyo, Japan; Sapporo Tsukisamu Chuo Orthopedics, Sapporo, Japan; Faculty of Health Science, Teikyo Heisei University, Ichihara, Japan; Institute for Integrated Sports Medicine, School of Medicine, Keio University, Tokyo, Japan; Orthopedics Surgery, School of Medicine, Jichi Medical University, Shimotsuke, Japan

**Keywords:** Topical NSAIDs, S-flurbiprofen, Pharmacokinetics, Tissue concentration, Synovial tissue

## Abstract

**Purpose:**

The purpose of this study is to investigate the pharmacokinetics and deep tissue penetration capability of the newly developed S-flurbiprofen plaster (SFPP) in humans.

**Methods:**

Study 1: SFPP tape-type patch (2–60 mg) was applied to the lower back for 24 h in healthy adult volunteers. S-flurbiprofen (SFP) plasma concentration was measured over time to examine SFP pharmacokinetics.

Study 2: SFPP (20 mg) was applied for 12 h to the affected knee of osteoarthritis (OA) patients who were scheduled for total knee arthroplasty. Deep tissues (synovial tissue and synovial fluid) were collected during surgery to compare SFP concentrations after application of SFPP or a commercially available flurbiprofen (FP) gel-type patch.

**Results:**

Study 1: The plasma concentration of SFP was sustained during 24-h topical application of the SFPP, showing a high percutaneous absorption ratio of 51.4–72.2 %. C_max_ and AUC_0-∞_ were dose-proportional.

Study 2: After application of the SFPP for 12 h, SFP concentrations in the synovial tissue and synovial fluid were 14.8-fold (*p* = 0.002) and 32.7-fold (*p* < 0.001) higher, respectively, than those achieved by the FP patch.

**Conclusions:**

Sustained plasma concentration of SFP and high percutaneous absorption ratio was observed after 24-h topical application of the SFPP. Compared to the FP patch, the SFPP showed superior percutaneous absorption and greater tissue penetration of SFP into the synovial tissue. Greater tissue penetration of the SFPP seemed to be primarily due to its formulation. Thus, SFPP is expected to show higher efficacy for the treatment of knee OA.

**Electronic supplementary material:**

The online version of this article (doi:10.1007/s00228-015-1960-6) contains supplementary material, which is available to authorized users.

## Introduction

Major symptoms of osteoarthritis (OA) are chronic pain and functional disorder derived from synovitis, both of which substantially decrease the quality of life of afflicted patients [1, 2]. Oral and topical nonsteroidal anti-inflammatory drugs (NSAIDs) have been widely used to treat the symptoms of OA and such use is recommended by various guidelines [3–5].

Because pain severity in OA patients is associated with the degree of synovitis [6, 7], it is important to confirm the drug concentration in synovial tissue when predicting the therapeutic effect of topical NSAIDs [8, 9]. Therefore, drug concentrations in knee joint tissue after application of topical NSAIDs have been determined [8–13].

We developed a new generation topical NSAID patch, the S-flurbiprofen plaster (SFPP). S-flurbiprofen (SFP) is an enantiomer of racemic flurbiprofen (FP), and SFP potently inhibits cyclooxygenase (COX)-1 and COX-2, whereas the corresponding inhibitory effects of R-flurbiprofen (RFP) are extremely weak [14, 15]. The commercially available gel-type FP patch, which consists of a water-soluble polymer and water, has low percutaneous absorption and relative bioavailability of about 4 % of that of the oral tablet [16]. The SFPP was developed in the form of a tape-type patch, consisting of a hydrophobic polymer without water, and SFP dissolved in minimal amount of solvent, to achieve highly improved percutaneous absorption and superior penetration into deep tissue. In animal studies, the SFPP produced high percutaneous absorption, penetration into deep inflammatory tissue, analgesic activity, and anti-inflammatory activity (submitted).

The present studies were aimed at elucidating the plasma pharmacokinetics and deep tissue penetration of the SFPP in humans. First, the pharmacokinetic profile of the SFPP after a single application to the lower back of healthy adult volunteers was examined (study 1). Next, concentrations of SFP in deep tissues (synovial tissue and synovial fluid) were examined in patients with knee OA scheduled for total knee arthroplasty and compared to those produced by a gel-type FP patch which contains the same amount of SFP and is commercially available in European companies, South Africa, and Japan for many years (study 2).

## Methods

### Test patches

SFPP tape-type patches containing 2, 5, 10, and 20 mg of SFP, placebo patches containing no SFP, and FP gel-type patches (Stayban^®^) containing 40 mg of FP were manufactured by Tokuhon Corporation (Tokyo, Japan). With the limitation of solubility of SFP, maximum SFP content was limited to 20 mg per patch (size 7 cm × 10 cm).

### Study design

#### Study 1: pharmacokinetic study in healthy volunteers

The study protocol was approved by the Institutional Review Board (IRB) of the Bio-Iatoric Center at Kitasato Institute (Tokyo, Japan) and conducted at the Bio-Iatoric Center at Kitasato Institute. The study was conducted according to the Declaration of Helsinki and Good Clinical Practice guidelines. Healthy adult male volunteers aged 20–35 years were included in the study. Those who had skin wounds, acne, or other abnormalities at the site of application (lower back) and those who had used any other drug within the week prior to the application of the study drug were excluded from the study.

SFPP tape-type patches (7 cm × 10 cm), containing 2, 5, 10, or 20 mg of SFP, were used in this study. To evaluate 6 dose regimens (2, 5, 10, 20, 40, and 60 mg), one sheet of 2-, 5-, 10-, or 20-mg patches was applied for the 2–20 mg dose regimens, whereas 2 and 3 sheets of 20-mg patches were applied for the 40-mg and 60-mg dose regimens, respectively. A placebo patch containing no SFP was also used as the comparator for safety evaluation.

**Fig. 1:**
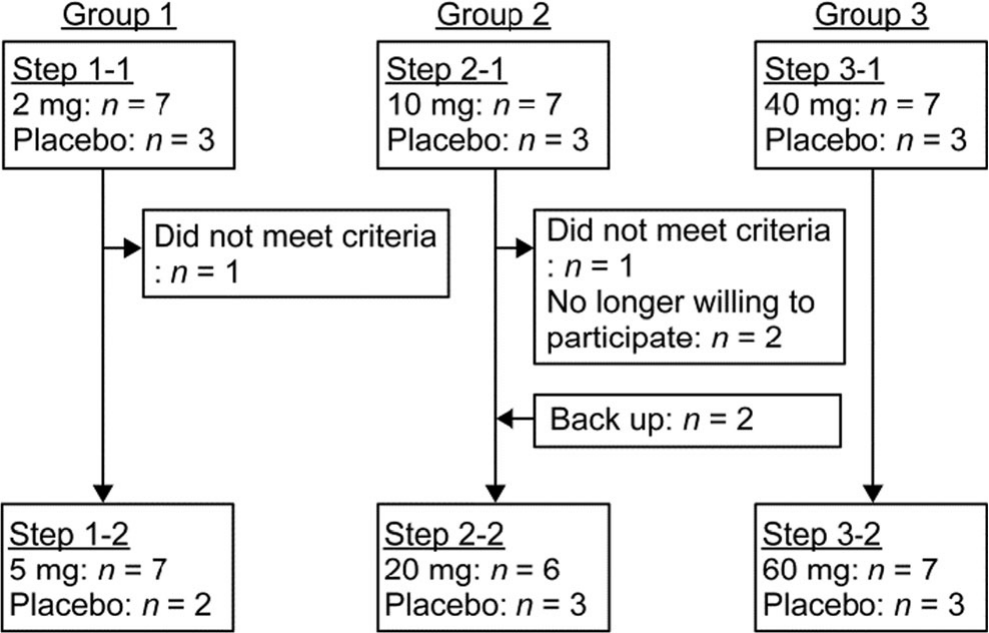
Flow chart of study 1

After written consent was obtained, 30 healthy male volunteers were enrolled in a randomized, single-blinded, placebo-controlled, dose-escalation phase 1 study. Each subject was applied two times at the interval of 2 weeks. The subjects were allocated to 3 groups of 10 persons each and were randomly given the SFPP (*n* = 7) or placebo (*n* = 3) at two steps (group 1 2 mg/5 mg; group 2 10 mg/20 mg; group 3 40 mg/60 mg) (Fig. 1).

The SFPP or placebo patch was applied to the lower back for 24 h. Blood samples were collected 1 h before application and at 2, 4, 6, 8, 10, 12, 14, 24, 28, 32, 36, 47, and 71 h after application (5 mL on each occasion). The collected blood samples were centrifuged immediately. Plasma samples were stored at approximately −20 °C until SFP concentrations were measured. The applied patches were collected to calculate the percutaneous absorption ratio.

#### Study 2: tissue concentration study for knee OA

The study protocol was approved by the IRB of Eniwa Hospital (Hokkaido, Japan), and the study was conducted at Eniwa Hospital. The study was conducted according to the Declaration of Helsinki and Good Clinical Practice guidelines.

The study included patients with knee OA scheduled for unilateral total knee arthroplasty. Patients who had a history of surgery (excluding arthroscopic-assisted surgery) of the target knee and those who had received FP or undergone arthroscopic examination, centesis, or drainage of the target knee within 7 days prior to the application of the study drug were excluded.

SFPPs (10 cm × 14 cm), containing 20 mg of SFP, were used in the study. The comparator FP patch was the Stayban^®^ pap (10 cm × 14 cm) gel-type patch containing 40 mg of FP. The SFP content was identical (20 mg) in the tested patches.

Twenty patients who gave written consent were included in this randomized open-label, parallel group comparative study. SFPPs and FP patches were cut in half (7 cm × 10 cm) and applied to the target knee at a site about 2 cm distant from the intended incision line on the right and left sides. SFPPs and FP patches were applied to 10 patients each, after which the SFP concentration in deep tissue of the knee and safety were evaluated.

The patches were applied for 12 h, which approximated t_max_ on the basis of the pharmacokinetic parameters of the FP patch [17] and the pharmacokinetic parameters of the SFPP determined in a single application study (study 1). Each patch was removed just before surgery. Synovial tissue, synovial fluid, and plasma were collected at the time of surgery and stored at approximately −20 °C until SFP concentrations were measured. The applied patches were collected to calculate the percutaneous absorption ratio.

### Analytical methods

#### Study 1

The SFP concentrations in the plasma and patches were determined by high-performance liquid chromatography (HPLC) at Sumika Chemical Analysis Service, Ltd. (Osaka, Japan). The validated SFP plasma concentration range was 2–800 ng/mL, intra-run precision was ≤4.8 %, and intra-run accuracy was −6.2 to 10.0 %. The validated SFP concentration range in a sheet of the patch was 0.2–25 mg, with intra-run precision of ≤2.4 % and intra-run accuracy of −9.5 to 5.0 % (further details are provided in supplementary documents).

#### Study 2

The SFP concentrations in tissue and plasma were determined by liquid chromatography and mass spectrometry (LC-MS/MS) and the SFP and FP concentrations in the patches were determined by HPLC at Taisho Pharmaceutical Co., Ltd. (Tokyo, Japan). The validated SFP concentration range was 5–10,000 ng/g for synovial tissue and 0.5–1000 ng/mL for synovial fluid and plasma. Intra-run precision and intra-run accuracy were ≤3.3 % and −2.5 to 6.1 % for synovial tissue, ≤ 4.4 % and −4.6 to 1.7 % for synovial fluid, and ≤4.7 % and 1.2 to 11.6 % for plasma. The range of validated drug concentrations in a patch sheet was 8–48 mg of SFP for the SFPP and 22.4–40 mg of FP for the FP patch. Intra-run precision and intra-run accuracy were ≤0.6 % and −0.4 to 1.6 % for the SFPP and ≤0.3 % and 0.9 to 1.9 % for the FP patch (further details are provided in supplementary documents).

### Pharmacokinetic analysis

Pharmacokinetic parameters were calculated as follows. C_max_ and t_max_ were obtained directly from the plasma concentration. The elimination rate constant (λz) was obtained by log-linear regression of the terminal phase of the plasma concentration-time curve. The t_1/2_ was calculated as ln2/λz. AUC_0-∞_ was calculated as the sum of the area from time zero to the last quantifiable time point *t* obtained by the trapezoidal method, and the area from *t* to infinite time calculated from Ct/λz; Ct is the last observed quantifiable concentration.

The percutaneous absorption ratio was calculated from the SFP amount in the patch (A) and the residual amount in the used patch (B) according to the following equation:

$$ \mathrm{Absorption}\ \mathrm{ratio}\left(\%\right)=\frac{\left(\mathrm{A}\hbox{--} \mathrm{B}\right)}{\mathrm{A}}\times 100 $$

### Statistical analysis

#### Study 1

Descriptive statistics for plasma concentrations, pharmacokinetic parameters, and absorption were calculated by dose group. Dose proportionality for C_max_ and AUC_0-∞_ was evaluated by determining whether the two-sided 95 % confidence intervals (CI) of regression coefficient estimates for these parameters did or did not include 1 using the power model.

#### Study 2

All analyses were carried out using SAS^®^ 9.1.3. Statistical comparisons between groups were made using Welch’s *t* test because it could be assumed that the absorption ratios achieved by the SFPP and FP patches were quite different. Therefore, below-the-limit-of-quantification (BLQ) data was imputed using the lower limit of quantification (LLOQ; 5.0 ng g^−1^ for synovial tissue and 0.500 ng mL^−1^ for synovial fluid and plasma) when the concentration was BLQ, to avoid skewing the results for the FP patch. The significance level was set at 5 % (two-sided).

## Results

### Study 1

In this study, 32 healthy adult volunteers (a cumulative total of 58 subjects at all steps in group 1, group 2, and group 3) were examined (Fig. 1). Table 1 shows the demographics of the subjects (cumulative total, 41) in whom the plasma SFP concentration was determined after application of the SFPP.

**Table 1:** Subject demographics (study 1)

	Group 1		Group 2		Group 3	
	2 mg (*n* = 7)	5 mg (*n* = 7)	10 mg (*n* = 7)	20 mg (*n* = 6)	40 mg (*n* = 7)	60 mg (*n* = 7)
Age (years)	23.0 ± 3.7	22.9 ± 2.5	22.3 ± 2.1	22.5 ± 1.9	24.1 ± 3.3	23.3 ± 2.3
	(20–30)	(20–26)	(20–26)	(21–26)	(21–30)	(21–26)
Weight (kg)	61.01 ± 8.07	61.93 ± 8.36	63.89 ± 6.70	61.48 ± 7.55	58.54 ± 4.37	60.39 ± 6.73
	(53.0–74.7)	(51.4–74.7)	(54.8–72.9)	(54.4–72.9)	(53.1–66.6)	(50.4–70.7)
BMI (kg m^−2^)	21.19 ± 2.07	21.06 ± 2.48	21.45 ± 1.95	20.97 ± 2.27	20.06 ± 0.82	20.25 ± 1.15
	(18.9–24.9)	(17.7–24.9)	(19.1–24.4)	(18.6–24.4)	(19.2–21.7)	(18.7–21.7)

Mean ± SD (range)

**Fig. 2:**
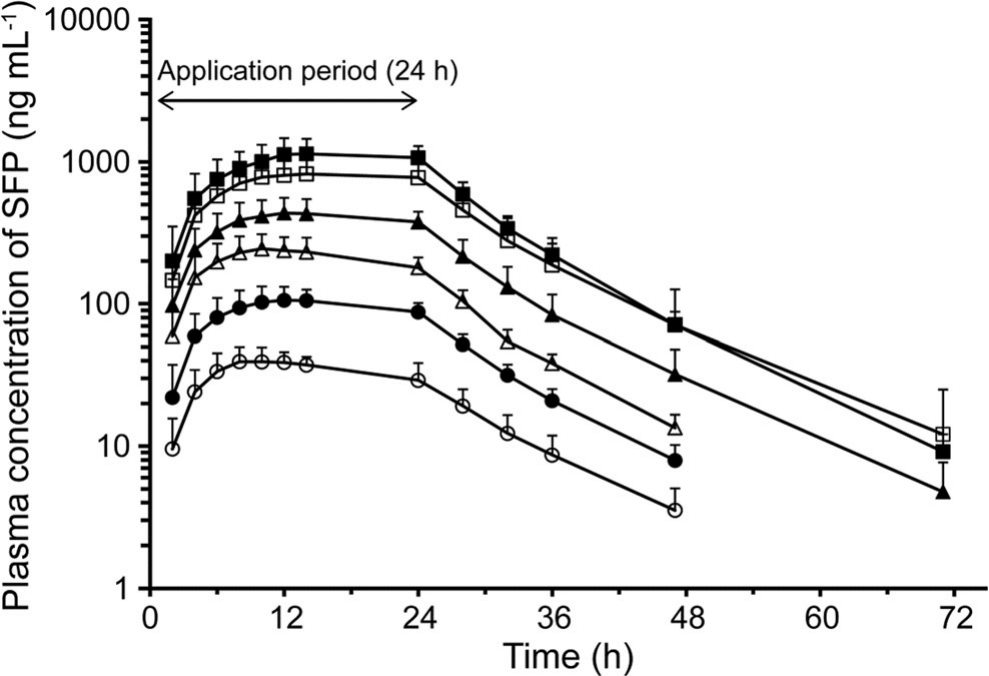
Plasma concentrations of SFP (mean + SD, log scale) vs. time profiles 24 h after topical application of the SFPP (2 mg (*white circle*), 5 mg (*filled circle*), 10 mg (*white triangle*), 20 mg (*filled triangle*), 40 mg (*white square*), and 60 mg (*filled square*))

The mean plasma concentrations vs. time profiles and pharmacokinetic parameters of SFP are shown in Fig. 2 and Table 2, respectively. The t_max_ and t_1/2_ values for the plasma SFP concentration were 10.3–17.7 h and 7.6–8.4 h, respectively. The percutaneous absorption ratio, determined based on the residual amount of SFP in the patch, was 51.4–72.2 %.

**Table 2:** Pharmacokinetic parameters and absorption ratios of SFP after topical application of the SFPP (24 h)

	2 mg (*n* = 7)	5 mg (*n* = 7)	10 mg (*n* = 7)	20 mg (*n* = 6)	40 mg (*n* = 7)	60 mg (*n* = 7)
C_max_ (ng mL^−1^)	43.3 ± 7.1	114.5 ± 20.3	248.0 ± 63.5	444.1 ± 120.3	858.2 ± 235.8	1187.7 ± 294.6
t_max_ (h)	13.7 ± 7.2	15.1 ± 6.2	10.3 ± 1.4	14.7 ± 4.7	17.7 ± 6.0	16.3 ± 5.3
t_1/2_ (h)	8.3 ± 0.9	7.8 ± 1.2	7.8 ± 1.1	8.4 ± 0.5	8.4 ± 1.1	7.6 ± 0.4
AUC_0-∞_ (ng mL^−1^ h)	1051.0 ± 156.7	2801.4 ± 414.2	6055.8 ± 1422.8	11,705.3 ± 3054.3	23,129.8 ± 7854.7	30,236.9 ± 7429.7
Absorption ratio (%)	61.2 ± 10.7	68.0 ± 12.4	72.2 ± 10.5	64.1 ± 10.5	57.5 ± 9.3	51.4 ± 7.9

Mean ± SD

The dose proportionality of C_max_ and AUC_0-∞_ was examined using a power model. Table 3 shows the regression coefficients (95 % CI) of C_max_ and AUC_0-∞_ at the dose range 2–20 mg, 20–60 mg, and 2–60 mg. For 2–20 mg, only the SFP content in the patches were different. For 20–60 mg, only the number of patch was different which means the application area was enlarged to increase the dose. The 95 % CI in each range includes 1, showing dose proportionality, respectively. In addition, although the application area and the SFP content of the patches were different, dose proportionality was confirmed at the dose range of 2–60 mg.

**Table 3:** Regression coefficients (95 % CI) of C_max_ and AUC_0−∞_

Dose range (mg)	Regression coefficients (95 % CI)	
	C_max_	AUC_0-∞_
2–20^1^	1.0175 (0.9154–1.1197)	1.0483 (0.9564–1.1402)
20–60^2^	0.9042 (0.6437–1.1647)	0.8719 (0.5926–1.1513)
2–60	0.9679 (0.9076–1.0282)	0.9910 (0.9303–1.0517)

^1^One sheet of 2-, 5-, 10-, or 20-mg patches

^2^1, 2, and 3 sheets of 20-mg patches

With regard to safety, cutaneous symptoms were the major adverse reactions found at the site of topical application in the SFPP (36.6 %, 15/41 subjects) and placebo groups (41.2 %, 7/17 subjects). Mild erythema was the most common cutaneous symptom (72.7 %, 16/22 subjects) and recovered promptly without any treatment.

### Study 2

This study included 20 patients with knee OA scheduled for total knee arthroplasty. One patient was excluded from pharmacokinetic analysis due to the prior use of the patch containing FP, which was prohibited. The subject demographics of each group are shown in Table 4.

**Table 4:** Subject demographics (study 2)

	SFPP (*n* = 10)	FP (*n* = 9)
Male/female	1/9	0/9
Age (years)	72.2 ± 6.4	72.0 ± 6.8
	(60–80)	(59–78)
Weight (kg)	69.23 ± 15.77	62.53 ± 3.66
	(42.7–102.8)	(57.1–69.3)
BMI (kg m^−2^)	29.74 ± 5.38	28.51 ± 1.67
	(23.0–41.2)	(26.1–31.0)

Mean ± SD (range)

Table 5 shows the SFP concentrations in each tissue and absorption ratios after topical application. Because the synovial fluid could not be collected in one of the subjects in the SFPP group, the calculation included 9 subjects. The SFP concentration in synovial tissue after application of the FP patch was BLQ in 8 of 9 subjects. Therefore, the LLOQ (5 ng g^−1^) was used in the calculation according to the statistical analysis plan.

**Table 5:** SFP concentrations in each tissue type and absorption ratios after topical application of the SFPP or FP patch (12 h)

	SFFP (*n* = 10)		FP (*n* = 9)		*P* value
Synovial tissue (ng g^−1^)	84.5 ± 56.0	(44.5, 125)	5.70 ± 2.10^a^	(−)^a^	0.002
Synovial fluid (ng mL^−1^)	149 ± 44.9^b^	(114, 183)	4.55 ± 3.66	(1.74, 7.37)	<0.001
Plasma (ng mL^−1^)	362 ± 84.8	(302, 423)	10.5 ± 10.1	(2.74, 18.3)	<0.001
Absorption ratio (%)	44.46 ± 10.63	(36.85, 52.07)	5.82 ± 1.64	(4.56, 7.08)	<0.001

Mean ± SD (95 % CI)

LLOQ, 5.0 ng g^−1^ for synovial tissue and 0.500 ng mL^−1^ for synovial fluid and plasma

^a^Eight samples of synovial tissue (FP) contained SFP below the limit of quantitation, so 5.0 ng g^−1^ (LLOQ) was used to calculate the mean and SD (95 % CI was not calculated)

^b^ *n* = 9 (one subject’s synovial fluid could not be collected)

The SFP concentrations in synovial tissue, synovial fluid, and plasma after topical application of the SFPP were significantly higher (*p* < 0.05) than those achieved by the FP patch, with mean values 14.8-, 32.7-, and 34.5-fold higher, respectively. The percutaneous absorption ratio of the SFPP was also significantly higher (*p* < 0.05) than that of the FP patch, with a mean value 7.6-fold higher.

With regard to safety, there were no adverse reactions in either group.

## Discussion

Plasma SFP concentrations in healthy adult volunteers after a single topical application of the SFPP for 24 h were dose proportional and sustained throughout the topical application period. The results showed that the SFPP has a favorable pharmacokinetic profile allowing once-daily application.

The formulation of topical NSAIDs is known to greatly affect their percutaneous absorption [18]. The SFPP and FP patch showed marked differences in percutaneous absorption and tissue penetration, which were likely primarily due to differences in the patch formulations. The SFPP is a tape-type patch, whereas the FP patch is a gel-type patch. It is presumed that high concentrations of lipophilic SFP dissolved in the patch (consisting of a hydrophobic polymer) contributed to the high percutaneous absorption and tissue penetration achieved by the SFPP. In addition, medium-chain fatty acid ester, a penetration enhancer [19], was used as an additive in the SFPP and may have contributed to the observed improvement in percutaneous absorption.

Bolten reported the FP concentrations in synovial fluid and serum after topical application of a gel-type FP patch in patients who required surgery for knee OA and orthopedic disease [10]. The reported data are tissue concentrations of FP; the ratio of SFP concentration to RFP concentration is unclear. However, considering that the ratio of SFP C_max_ to RFP C_max_ after oral administration of FP was 1.05 [20], and there was no chiral inversion of enantiomers [21], the SFP concentration was estimated as half of the FP concentration. According to this estimation, the SFP concentrations in synovial fluid and plasma 12 h after topical application of the FP patch in our study were similar to those reported by Bolten.

C_max_ and AUC_0-∞_ after oral administration of SFP 50 mg in gelatin capsule were reported as 9.3 μg mL^−1^ and 55.2 μg h mL^−1^, respectively [21]. As dose proportionality was observed in the SFPP, C_max_ and AUC_0-∞_ after application of the SFPP 50 mg could be proportionally calculated from those of the SFPP 40 mg and 60 mg as 1.0 μg mL^−1^ and 26.7 μg h mL^−1^. The pharmacokinetic parameters ratios (SFP oral administration/SFPP) are approximately 9 for C_max_ and 2 for AUC_0-∞_, which mean smaller values are observed in the SFPP. It was reported that the plasma concentration after repeated application of FP patch was 2.5 times higher than that after single application [16]. So it must be important to study the pharmacokinetics after repeated application of SFPP.

In this study, we measured SFP concentrations in synovial tissue and synovial fluid at a single time point around t_max_ (12 h after topical application), without conducting measurements at other time points. Because plasma SFP concentrations were similar 12 and 24 h after topical application of the SFPP for 24 h, it is speculated that the SFP concentrations in synovial tissue and synovial fluid persist in the same manner as the plasma SFP concentration.

In conclusion, topical application of the SFPP for 24 h provided a persistent high plasma concentration of SFP in a dose-proportional manner. In addition, the SFP concentrations in synovial tissue and synovial fluid after application of the SFPP, as well as the percutaneous absorption ratio of SFP, were significantly higher than those after application of the FP patch. It is expected that the SFPP may exert a potent anti-inflammatory and analgesic effect by achieving penetration of a high concentration of SFP into deep synovial tissue, the site of inflammation in patients with knee OA.

## Electronic Supplementary Material

ESM 1 (DOCX 15.8 kb)
