# Supplementary material for: Plasma pharmacokinetics and synovial concentrations of S-flurbiprofen plaster in humans
Source: Eur J Clin Pharmacol. 2015 Oct 6;72:53–9. doi: 10.1007/s00228-015-1960-6 (PMC4701782; doi:10.1007/s00228-015-1960-6)
Supplement: Supplementary file 1 — (DOCX 15.8 kb) [file 228_2015_1960_MOESM1_ESM.docx]

**supplementary document**

**Analytical methods**

*Study 1*

The SFP concentrations in the plasma and patches were determined by high-performance liquid chromatography (HPLC) at Sumika Chemical Analysis Service, Ltd. (Osaka, Japan). The plasma samples were mixed with citrate buffer (pH 2.4) and 4-biphenylacetic acid (the internal standard [I.S.]) and extracted with chrolobutane/butanol (4/1, v/v). The organic phase was evaporated to dryness under a stream of nitrogen gas. The residue was dissolved in mobile phase A (acetonitrile/0.01 mol/L acetic acid [1/1, v/v]), filtered, and analyzed by HPLC. The analytical column was a Shim-Pack CLC-ODS (4.6 mm × 150 mm; Shimadzu, Kyoto, Japan). SFP was detected by a fluorescence detector (excitation wavelength: 262 nm; fluorescence wavelength: 313 nm) with mobile phase A run at a flow rate of 1 mL/min. The validated plasma concentration range was 2–800 ng/mL, intra-run precision was ≤ 4.8%, and intra-run accuracy was -6.2 to 10.0%.

Each patch was minced, immersed in acetone, and subjected to stirring after addition of the I.S. The supernatant was isolated and evaporated to dryness under a stream of nitrogen gas. The residue was dissolved in mobile phase B (methanol/water [65/35, v/v, adjusted to pH 3.0 with phosphoric acid]) and analyzed by HPLC. The analytical column was a Shiseido Capcell Pak C18 UG120 S-5 (4.6 mm × 150 mm; Shiseido, Tokyo, Japan) with mobile phase B run at a flow rate of 1 mL/min. SFP was detected with a fluorescence detector (excitation wavelength: 262 nm; fluorescence wavelength: 313 nm). The validated SFP concentration range in a sheet of the patch was 0.2–25 mg, with intra-run precision of ≤ 2.4% and intra-run accuracy of -9.5 to 5.0%.

*Study 2*

In this study, SFP and FP concentrations were measured by Taisho Pharmaceutical Co., Ltd. (Tokyo, Japan). SFP concentrations in tissue and plasma were determined by liquid chromatography and mass spectrometry (LC-MS/MS). Synovial tissue was homogenized after the addition of ice-cold distilled water. Water/acetic acid (98/2, v/v) and stable isotope-labelled flurbiprofen (flurbiprofen-d5; the I.S.) were added to the synovial tissue homogenate, synovial fluid, and plasma, which were subsequently extracted with hexane. The organic phase was evaporated to dryness under a stream of nitrogen gas. The residue was dissolved in mobile phase C (ammonium acetate/acetic acid/acetonitrile/water [0.482/0.625/900/100, w/v/v/v]) and analyzed by LC-MS/MS. Chromatography was performed with a YMC CHIRAL γ-CD BR column (2.0 mm × 250 mm; YMC, Kyoto, Japan) with mobile phase C (flow rate: 0.2 mL/min). An API4000 mass spectrometer (AB Sciex, Foster City, CA, USA) in negative ion mode was used for MS determination. Multiple reaction monitoring (MRM) transitions were *m/z* 243 to 199 for SFP and *m/z* 248 to 204 for the I.S. The validated SFP concentration range was 5–10000 ng/g for synovial tissue and 0.5–1000 ng/mL for synovial fluid and plasma. Intra-run precision and intra-run accuracy were ≤ 3.3% and -2.5 to 6.1%, respectively, for synovial tissue, ≤ 4.4% and -4.6 to 1.7%, respectively, for synovial fluid, and ≤ 4.7% and 1.2 to 11.6%, respectively, for plasma.

The concentrations of SFP and FP in the patches were measured by HPLC. The SFPP was minced, immersed in acetone, and subjected to stirring after the addition of diphenyl (the I.S.). The supernatant was used as the sample. The FP patch was also minced, immersed in a mixture of the I.S. and methanol, and subjected to stirring. The supernatant was used as the sample. Each sample was analyzed by HPLC. A Shiseido Capcell Pak C18 UG120 S-5 (4.6 mm × 150 mm) was used as the analytical column, with a mobile phase of methanol/water (7:3, v/v, adjusted to pH 3.0 with phosphoric acid) run at a flow rate of 1 mL/min. SFP and FP were detected with a UV detector (detection wavelength: 247 nm).

The range of validated drug concentrations in a patch sheet was 8–48 mg of SFP for the SFPP and 22.4–40 mg of FP for the FP patch. Intra-run precision and intra-run accuracy were ≤ 0.6% and -0.4 to 1.6%, respectively, for the SFPP and ≤ 0.3% and 0.9 to 1.9%, respectively, for the FP patch.
